# Bivalent Omicron BA.4/BA.5 BNT162b2 Vaccine in 6-Month- to <12-Year-Olds

**DOI:** 10.1093/jpids/piae062

**Published:** 2024-06-11

**Authors:** Lawrence D Sher, Justice K Boakye-Appiah, Sungeen Hill, Emily Wasserman, Xia Xu, Yvonne Maldonado, Emmanuel B Walter, Flor M Muñoz, Grant C Paulsen, Janet A Englund, Kawsar R Talaat, Elizabeth D Barnett, Satoshi Kamidani, Shelly Senders, Eric A F Simões, Kelly Belanger, Vrunda Parikh, Hua Ma, Xingbin Wang, Claire Lu, David Cooper, Kenneth Koury, Annaliesa S Anderson, Özlem Türeci, Uğur Şahin, Kena A Swanson, William C Gruber, Alejandra Gurtman, Nicholas Kitchin, Charu Sabharwal

**Affiliations:** Peninsula Research Associates, Rolling Hills Estates, California, USA; Vaccine Research and Development, Pfizer Ltd, Hurley, UK; Vaccine Research and Development, Pfizer Ltd, Hurley, UK; Vaccine Research and Development, Pfizer Inc, Pearl River, New York, USA; Vaccine Research and Development, Pfizer Inc, Collegeville, Pennsylvania, USA; Pediatric Infectious Disease, Stanford University School of Medicine, Palo Alto, California, USA; Duke Human Vaccine Institute, Duke University School of Medicine, Durham, North Carolina, USA; Pediatrics Infectious Disease, Texas Children’s Hospital, Baylor College of Medicine, Houston, Texas, USA; Department of Pediatrics, University of Cincinnati College of Medicine and Division of Pediatric Infectious Diseases, Cincinnati Children’s Hospital Medical Center, Cincinnati, Ohio, USA; Department of Pediatrics, Seattle Children’s Hospital, Seattle, Washington, USA; International Health, Johns Hopkins University, Baltimore, Maryland, USA; Department of Pediatrics, Boston Medical Center, BU Chobanian & Avedisian School of Medicine, Boston, Massachusetts, USA; The Center for Childhood Infections and Vaccines of Children’s Healthcare of Atlanta and the Department of Pediatrics, Emory University School of Medicine, Atlanta, Georgia, USA; Senders Pediatrics, South Euclid, Ohio, USA; Department of Pediatrics, Children’s Hospital Colorado, University of Colorado School of Medicine, Aurora, Colorado, USA; Samshoma Medical Research Inc., Denver, Colorado, USA; Vaccine Research and Development, Pfizer Inc, Pearl River, New York, USA; Vaccine Research and Development, Pfizer Inc, Pearl River, New York, USA; Vaccine Research and Development, Pfizer Inc, Collegeville, Pennsylvania, USA; Vaccine Research and Development, Pfizer Inc, Collegeville, Pennsylvania, USA; Vaccine Research and Development, Pfizer Inc, Pearl River, New York, USA; Vaccine Research and Development, Pfizer Inc, Pearl River, New York, USA; Vaccine Research and Development, Pfizer Inc, Pearl River, New York, USA; Vaccine Research and Development, Pfizer Inc, Pearl River, New York, USA; BioNTech, Mainz, Germany; BioNTech, Mainz, Germany; Vaccine Research and Development, Pfizer Inc, Pearl River, New York, USA; Vaccine Research and Development, Pfizer Inc, Pearl River, New York, USA; Vaccine Research and Development, Pfizer Inc, Pearl River, New York, USA; Vaccine Research and Development, Pfizer Ltd, Hurley, UK; Vaccine Research and Development, Pfizer Inc, Pearl River, New York, USA

**Keywords:** children, COVID-19, immunogenicity, safety, vaccination

## Abstract

**Background:**

With the future epidemiology and evolution of severe acute respiratory syndrome coronavirus 2 (SARS-CoV-2) uncertain, the use of safe and effective coronavirus disease 2019 (COVID-19) vaccines in pediatric populations remains important.

**Methods:**

We report data from two open-label substudies of an ongoing phase 1/2/3 master study (NCT05543616) investigating the safety and immunogenicity of a variant-adapted bivalent COVID-19 vaccine encoding ancestral and Omicron BA.4/BA.5 spike proteins (bivalent BNT162b2). The open-label groups presented here evaluate dose 4 with bivalent BNT162b2 in 6-month- to <12-year-olds who previously received three original (monovalent) BNT162b2 doses. In 6-month- to <5-year-olds, primary immunogenicity objectives were to demonstrate superiority (neutralizing titer) and noninferiority (seroresponse rate) to Omicron BA.4/BA.5 and noninferiority (neutralizing titer and seroresponse rate) to SARS-CoV-2 ancestral strains in participants who received bivalent BNT162b2 dose 4 compared with a matched group who received three doses of original BNT162b2 in the pivotal pediatric study (NCT04816643). In 5- to <12-year-olds, primary immunogenicity comparisons were descriptive. Reactogenicity and safety following vaccination were evaluated.

**Results:**

In 6-month- to <5-year-olds, dose 4 with bivalent BNT162b2 met predefined immunogenicity superiority and noninferiority criteria against Omicron BA.4/BA.5 and ancestral strains when compared with dose 3 of original BNT162b2. In 5- to <12-year-olds, bivalent BNT162b2 induced robust Omicron BA.4/BA.5 and ancestral strain neutralizing titers comparable with dose 3 of original BNT162b2. The safety profile for dose 4 of bivalent BNT162b2 given as dose 4 was consistent with that of original BNT162b2 in 6-month- to <12-year-olds. Reactogenicity events were generally mild to moderate. No adverse events led to discontinuation.

**Conclusions:**

These safety and immunogenicity data support a favorable benefit-risk profile for a variant-adapted BNT162b2 in children <12 years old.

## Teaser

The use of safe and effective COVID-19 vaccines in pediatric populations remains important. In this clinical trial, safety and immunogenicity data support the favorable benefit-risk profile for a variant-adapted bivalent BNT162b2 in children 6 months to 12 years old.

## INTRODUCTION

As of the end of April 2023, almost 17 million coronavirus disease 2019 (COVID-19) cases in the United States among <18-year olds, almost 60% were in children <12 years old [1]. Although children tend to have milder COVID-19 symptoms compared with adults [2], severe disease, including hospitalization, intensive care unit admission, mechanical ventilation, and death, can occur, particularly in <2-year-old children [3, 4]. A study conducted in the United States from August 2021 to July 2022 found that among 0- to 19-year-olds, COVID-19 was the fifth leading cause of disease-related deaths and the leading cause of infectious and respiratory disease-related death [5]. Peaks of COVID-19, influenza, and respiratory syncytial virus illnesses may overlap, particularly in winter [6]. Coinfection with other pathogens with or immediately after COVID-19 is estimated to occur in up to one in four of all COVID-19 patients [7]; such coinfections in children may be associated with poor outcomes [8, 9]. Although vaccine-derived and infection-acquired immunity have been observed across all ages, vaccination rates are low in children and lowest in infants and children <2 years old in the United States [10]; therefore, many may remain susceptible to severe acute respiratory syndrome coronavirus 2 (SARS-CoV-2) infection and potential complications of coinfection [11]. Using safe and effective COVID-19 vaccines in young children therefore remains important.

The original monovalent BNT162b2 COVID-19 mRNA vaccine was approved in 2021 as a two-dose primary series in ≥12-year-olds (30 μg) [12] and was subsequently authorized as a two-dose primary series for 5- to 11-year-old children (10 μg) in 2021, and as a three-dose primary series for 6-month- to <5-year-olds (3 μg) in 2022 [13, 14, 15]. With the emergence of variants differing substantially from the vaccine-encoded strain [16], a variant-adapted bivalent BNT162b2 vaccine comprising ancestral SARS-CoV-2 and Omicron BA.4/BA.5 spike proteins was authorized in late 2022 and recommended in place of the original BNT162b2 from 6 months of age in early 2023 [17]. Here we investigated the safety and immunogenicity of this variant-adapted bivalent Omicron BA.4/BA.5 plus original SARS-CoV-2 BNT162b2 (hereafter bivalent BNT162b2) as a fourth dose (after three doses of original BNT162b2) in 6-month- to <12-year-olds.

## METHODS

### Participants and Oversight

This report includes interim data from two open-label substudies from an ongoing phase 1/2/3 master study (Study C4591048) investigating safety, tolerability, and immunogenicity of bivalent BNT162b2 in healthy infants and children; the two phase 3 substudies included 6-month- to <5-year-old and 5- to <12-year-old participants, respectively. Bivalent BNT162b2 uses the same lipid-nanoparticle formulation and nucleoside-modified mRNA platform as original BNT162b2 with equal amounts of mRNA encoding both ancestral and Omicron BA.4/BA.5 complete spike proteins. The substudies were initiated in September 2022, when Omicron BA.4/BA.5 was the dominant circulating strain in the United States.

Healthy immunocompetent participants with or without evidence of previous SARS-CoV-2 infection were enrolled, including those with preexisting stable disease (ie, not requiring significant therapy modification or hospitalization for worsening disease during the 6 weeks before enrollment). Participants had received three previous doses of 3 μg (6-month- to <5-year-olds) or 10 μg (5- to <12-year-olds) BNT162b2, with the third dose administered 60–240 days (6-month- to <5-year-olds) or 90–240 days (5- to <12-year-olds) before study enrollment. Key exclusion criteria included history or current diagnosis of multisystem inflammatory syndrome in children (MIS-C), receipt of any medication intended to prevent COVID-19 or any COVID-19 vaccine other than BNT162b2, history of severe adverse reaction to a vaccine, anaphylaxis, or immunocompromise (see Supplementary Appendix for additional eligibility criteria).

Pfizer was responsible for study conduct; data collection, analysis and interpretation; and manuscript preparation. Pfizer and BioNTech were responsible for vaccine manufacture and study design. BioNTech sponsored the study and contributed to data interpretation and manuscript preparation. Authors had access to all data and vouch for study protocol adherence and data accuracy and completeness. The Supplementary Appendix has details on ethical conduct.

### Procedures

Participants received a single intramuscular dose of bivalent BNT162b2 into the deltoid muscle for those ≥2 years old, and into the anterior thigh muscle for those <2 years old. Infants and toddlers 6 months to <5 years old received 3 μg bivalent BNT162b2; 5- to <12-year-old children received 10 μg bivalent BNT162b2.

### Immunogenicity

Immunogenicity endpoints included SARS-CoV-2 Omicron BA.4/BA.5 and ancestral strain neutralization responses before and 1 month after dose 4 tested with a validated SARS-CoV-2 neutralization assay. Because Omicron BA.4 and BA.5 spike sequences are identical, a single recombinant SARS-CoV-2 virus expressing the Omicron BA.4/BA.5 spike was used in the neutralization assay. Additional neutralization testing was conducted in a subset of 6-month- to <5-year-olds (*n* = 31) to characterize Omicron BA.4/BA.5, XBB.1.5, and BQ.1.1 responses using a non-validated fluorescent focus reduction neutralization test [18, 19]. Such neutralization testing was not conducted in 5- to <12-year-old participants. Comparator groups for immunogenicity assessments were participants from the pivotal BNT162b2 pediatric study (C4591007 [NCT04816643]) who were matched by age, baseline SARS-CoV-2 infection status (6-month- to <12-year-olds), and time since previous dose (6-month- to <5-year-olds only). Comparator participants had received three original BNT162b2 3 μg (6-month- to <5-year-olds) or 10 µg (5- to <12-year-olds) doses with immunogenicity assessments conducted before and 1 month after dose 3.

Geometric mean ratios (GMRs) and differences in percentages of participants with seroresponse (defined in the Supplementary Appendix) between the bivalent BNT162b2 group and the comparator group were evaluated. Geometric mean titers (GMTs), geometric mean-fold rises (GMFRs), and percentages of participants with seroresponse at each time point following vaccination were also reported.

### Safety

Safety evaluations included monitoring for local reactions and systemic events by participants or their parents/guardians and reporting via electronic diary for 7 days after study vaccination (see Supplementary Table 1 for severity grading of local reactions and systemic events). Adverse events (AEs) and serious AEs (SAEs) occurring between 1 month and 6 months after vaccination are reported. Myocarditis and pericarditis occurring within 4 weeks after vaccination were designated AEs of special interest (monitoring of potential cases are described in the Supplementary Appendix).

### COVID-19 and MIS-C Surveillance

Surveillance for potential cases of COVID-19 and MIS-C is ongoing throughout the study.

### Statistical Analysis

Statistical hypothesis testing related to immunogenicity assessment in 6-month- to <5-year-old participants were conducted in the per-protocol set (Supplementary Table 2), a randomly selected subset representing the same distribution of age subgroups and baseline SARS-CoV-2 status as in the full set. Total sample sizes for both age groups were based on the safety database.

The evaluable immunogenicity population included all eligible participants who received bivalent BNT162b2 as the fourth dose (as assigned), had ≥1 valid and determinate immunogenicity result from the blood sample collected within an appropriate window after the fourth dose, and had no other important protocol deviations. Calculations of GMTs, model-adjusted GMRs, GMFRs, seroresponse, and adjusted differences in seroresponse are described in the Supplementary Appendix.

In 6-month- to <5-year-olds, primary immunogenicity objectives were to demonstrate superiority, with respect to neutralizing titer, and noninferiority, with respect to seroresponse rate, at 1 month after dosing, for Omicron BA.4/BA.5 immune responses in participants who received bivalent BNT162b2 (dose 4) compared with the matched comparator group who received 3 doses of original BNT162b2. The secondary objectives were to demonstrate noninferiority with respect to neutralizing titer and seroresponse rates for the ancestral strain. Omicron BA.4/BA.5 neutralizing titer superiority was defined as a lower bound of the GMR 95% CI of >1; noninferiority of seroresponse was defined as a lower bound of the 95% CI for the percentage difference in seroresponse of greater than −5%. Noninferiority criteria for the ancestral strain were a lower bound of neutralizing titer GMR 95% CI of >0.67 (and GMR point estimate ≥0.8) and lower bound of the 95% CI for the percentage difference in seroresponse of greater than −10%. Immunogenicity objectives were evaluated sequentially: superiority and noninferiority to Omicron BA.4/BA.5 followed by noninferiority to ancestral strain. Within each objective, GMR and seroresponse differences were assessed sequentially; both hypotheses within an objective were established before assessing the next, thus fully controlling overall type I error. In 5- to <12-year-olds, the primary immunogenicity comparisons were descriptive. Safety data are presented descriptively by age group (6-month- to <2-year-olds; 2- to <5-year-olds; 5- to <12-year-olds) as counts, percentages, and associated Clopper-Pearson two-sided 95% CIs. AEs were categorized according to the Medical Dictionary for Regulatory Activities v25.1 terminology.

## RESULTS

### Participants

From September 23, 2022, to February 1, 2023, for 6-month- to <5-year-olds and October 21, 2022, for 5- to <12-year-olds, 310 participants 6 months to <5 years old and 113 participants 5- to <12 years old received dose 4 with bivalent BNT162b2 (Supplementary Figure 1). The study was conducted at 19 sites in the United States. Participant demographic characteristics in the overall population of participants 6 months to <5 years old and 5 to <12 years old are shown in Supplementary Table 3. Among 6-month- to <5-year-olds, 50.3% of participants were male, 70.0% White, 2.9% Black, and 16.8% Hispanic/Latino. In 5- to <12-year-olds, 50.4% were male, 58.4% White, 8.0% Black, and 20.4% Hispanic/Latino. Serological/virological evidence of previous SARS-CoV-2 infection (ie, positive N-binding antibody result or positive nucleic acid amplification test result before receipt of bivalent BNT162b2, or medical history of COVID-19) was more common among 5- to <12-year-old participants (58.4%) than among 6-month- to <5-year-olds (40.3%). Median (range) time from dose 3 of original BNT162b2 to receipt with dose 4 of bivalent BNT162b2 was 6.7 (2.1–8.6) and 5.5 (2.6–8.5) months and corresponding median (range) follow-up time after vaccination was 6.3 (1.0–7.5) and 6.3 (1.1–6.8) months at data cutoff in 6-month- to <5-year-olds and 5- to <12-year-olds, respectively. Participant demographic characteristics for the evaluable population of participants 6 months to <5 years old and the associated comparator group are shown in Table 1 (and Supplementary Tables 4 and 5 for additional immunogenicity populations and comparator groups).

**Table 1.** Demographic Characteristics of Evaluable Immunogenicity Population 6 Months to <5 Years of Age Who Received Bivalent BNT162b2 in the Current Study and Immunogenicity Comparator Group of Participants 6 Months to <5 Years of Age Who Received Original BNT162b2 in the Original Pediatric Study

Characteristic	Received Bivalent BNT162b2 in Current Study			Received Original BNT162b2 in Original Pediatric Study		
	Evaluable Immunogenicity Population			Immunogenicity Comparator Group^a^		
	6 Months to <2 Years (*n* = 78)	2- to <5 Years (*n* = 196)	6 Months to <5 Years (*n* = 274)	6 Months to <2 Years (*n* = 92)	2- to <5 Years (*n* = 217)	6 Months to <5 Years (*n* = 309)
Sex, *n* (%)						
Male	43 (55.1)	93 (47.4)	137 (50.0)	48 (52.2)	101 (46.5)	149 (48.2)
Female	35 (44.9)	103 (52.6)	137 (50.0)	44 (47.8)	116 (53.5)	160 (51.8)
Race or ethnicity, *n* (%)						
White	56 (71.8)	135 (68.9)	191 (69.7)	70 (76.1)	166 (76.5)	236 (76.4)
Black	1 (1.3)	7 (3.6)	8 (2.9)	4 (4.3)	13 (6.0)	17 (5.5)
Asian	8 (10.3)	16 (8.2)	24 (8.8)	9 (9.8)	17 (7.8)	26 (8.4)
Multiracial	13 (16.7)	38 (19.4)	51 (18.6)	8 (8.7)	20 (9.2)	28 (9.1)
Not reported/other	0	0	0	1 (1.1)	1 (0.5)	2 (0.6)
Hispanic/Latino	17 (21.8)	32 (16.3)	49 (17.9)	14 (15.2)	31 (14.3)	45 (14.6)
Age at study vaccination						
Mean (standard deviation)	19.4 (3.29) months	2.9 (0.84) years	N/A	19.1 (3.33) months	2.9 (0.84) years	N/A
Median (range)	20 (11–23) months	3 (2–4) years	N/A	20 (9–23) months	3 (2–4) years	N/A
Median time (range) since previous dose of original BNT162b2, months	5.3 (2.1, 8.6)	7.0 (2.2, 8.6)	6.8 (2.1, 8.6)	6.1 (2.2, 8.8)	7.0 (2.1, 9.1)	6.8 (2.1, 9.1)
Baseline SARS-CoV-2 status,^b^*n* (%)						
Positive	36 (46.2)	76 (38.8)	112 (40.9)	44 (47.8)	83 (38.2)	127 (41.1)
Negative	38 (48.7)	117 (59.7)	155 (56.6)	48 (52.2)	133 (61.3)	181 (58.6)
Missing	4 (5.1)	3 (1.5)	7 (2.6)	0	1 (0.5)	1 (0.3)
Comorbidities,^c^*n* (%)	6 (7.7)	24 (12.2)	30 (10.9)	4 (4.3)	21 (9.7)	25 (8.1)

Abbreviations: BMI, body mass index; NA, not applicable; NAAT, nucleic acid amplification test; N-binding, SARS-CoV-2 nucleoprotein-binding.

^a^Participants in the original pediatric study (NCT04816643) who were 6 months to <5 years old and who had received three doses of original BNT162b2 10 μg.

^b^According to N-binding antibody or NAAT result at the study vaccination visit (ie, dose 4 with bivalent BNT162b2 for study participants), and medical history of COVID-19. See the Supplementary Appendix for the definitions of the immunogenicity comparator group.

^c^Number of participants who had ≥1 comorbidity that increases the risk of severe COVID-19: defined as participants who had ≥1 of the prespecified comorbidities as defined by Kim et al [20] (6 months to <12 years of age), and the Centers for Disease Control and Prevention [21] (6 months to <5 years of age), and/or obesity (BMI ≥95th percentile; 2 to <12 years of age). Comorbidities were assessed at the first study visit for both studies.

### Immunogenicity

**Figure 1.**
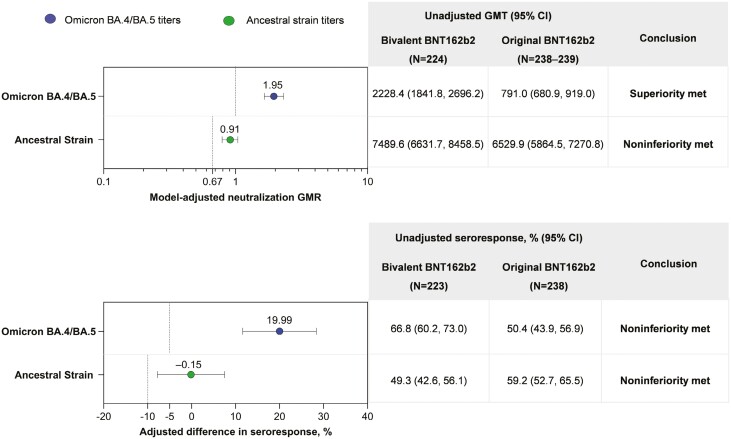
(A) Model-adjusted GMRs and unadjusted GMTs and (B) adjusted differences in percentages of participants with seroresponses 1 month after bivalent BNT162b2 (dose 4) and original BNT162b2 (dose 3) in participants 6 months to <5 years of age. Data are for the per-protocol set of the evaluable immunogenicity population (see Supplementary Table 2) and include participants with and without evidence of previous SARS-CoV-2 infection. Data for original BNT162b2 are in participants from the original pediatric study (NCT04816643) who were matched by age, baseline SARS-CoV-2 status, and time since previous BNT162b2 dose (see Table 1 for demographic details for these participants and those of the evaluable immunogenicity population of the current study). Shown in (A) are model-adjusted GMRs (95% CIs) of the vaccine comparison (graph) and associated unadjusted GMTs (table). Assay results below the LLOQ were set to 0.5 × LLOQ. Shown in (B) are the adjusted differences in percentages (95% CIs) of participants with Omicron BA.4/BA.5 and ancestral strain seroresponses and associated unadjusted seroresponse rates. Dotted lines represent superiority or noninferiority criteria for each comparison. Omicron BA.4/BA.5 neutralizing titer superiority was defined as a lower bound of the GMR 95% CI of >1; noninferiority of seroresponse was defined as lower bound of the 95% CI for the percentage difference in seroresponse of > −5%. Noninferiority criteria for the ancestral strain were a lower bound of neutralizing titer GMR 95% CI of >0.67 (and GMR point estimate ≥0.8) and a lower bound of the 95% CI for the percentage difference in seroresponse of > −10%. GMR, geometric mean ratio; GMT, geometric mean titer; LLOQ, lower limit of quantitation.

Among evaluable 6-month- to <5-year-old participants, the model-adjusted GMR of 50% neutralizing titers against Omicron BA.4/BA.5 in the bivalent BNT162b2 group (dose 4) compared with the original BNT162b2 group (dose 3) was 1.95 (95% CI, 1.65, 2.31), thus meeting the predefined superiority criterion (Figure 1A). The adjusted difference in the percentage of participants with seroresponse to Omicron BA.4/BA.5 between the original BNT162b2 group and the bivalent BNT162b2 group was 19.99% (95% CI, 11.61, 28.36), meeting the predefined noninferiority criterion (Figure 1B). When participants were stratified by age (6-month- to <2-year-olds and 2- to <5-year-olds), consistent model-adjusted GMRs of 50% neutralizing titers against Omicron BA.4/BA.5 in the bivalent BNT162b2 group compared with the original BNT162b2 group (1.61 [95% CI, 1.20, 2.18] and 2.13 [95% CI, 1.73, 2.62], respectively), and differences in seroresponse rates (16.24% [95% CI, 0.80, 31.67] and 21.37% [95% CI, 11.59, 31.15], respectively) were observed (Supplementary Table 6).

Among evaluable 6-month- to <5-year-old participants, the model-adjusted GMR of 50% neutralizing titers against the ancestral strain was 0.91 (95% CI, 0.79, 1.04), meeting the predefined noninferiority criterion. The adjusted differences in percentage of participants with seroresponse to the ancestral strain between the original BNT162b2 group and the bivalent BNT162b2 group was −0.15% (95% CI, −7.79, 7.48), meeting the predefined noninferiority criterion.

In the descriptive analysis in 5- to <12-year-olds, the model-adjusted GMR of Omicron BA.4/BA.5 neutralizing titers for the bivalent BNT162b2 group to the original BNT162b2 group was 1.12 (95% CI: 0.92, 1.37; Supplementary Table 7) and the adjusted difference in percentages of participants with seroresponse to Omicron BA.4/BA.5 between the bivalent BNT162b2 and original BNT162b2 groups was 8.76% (95% CI: −2.47, 19.99; Supplementary Table 8).

SARS-CoV-2 neutralization assay results before and 1 month after vaccination in all evaluable participants are shown in Supplementary Figure 2. Among 6-month- to <5-year-olds, within both baseline SARS-CoV-2-positive and baseline SARS-CoV-2-negative groups, GMTs against Omicron BA.4/BA.5 were higher in the bivalent BNT162b2 group compared with the original BNT162b2 group at prevaccination and more substantially 1 month after vaccination. For 5- to <12-year-olds with evidence of prior infection, BA.4/BA.5 neutralizing GMTs at 1 month after vaccination were higher in the bivalent BNT162b2 group compared with the original BNT162b2 group (3465.6 vs 1893.9). For 5- to <12-year-olds without evidence of prior infection, observed GMTs at 1 month after vaccination were numerically higher in the bivalent BNT162b2 (1195.8) and original BNT162b2 (905.8) groups; although the differences were more subtle, increased Omicron BA.4/BA.5 responses were consistently observed regardless of baseline SARS-CoV-2 status. In both age groups, ancestral strain-specific neutralizing titers were generally similar 1 month after vaccination in the bivalent BNT162b2 group compared with the original BNT162b2 group.

**Figure 2.**
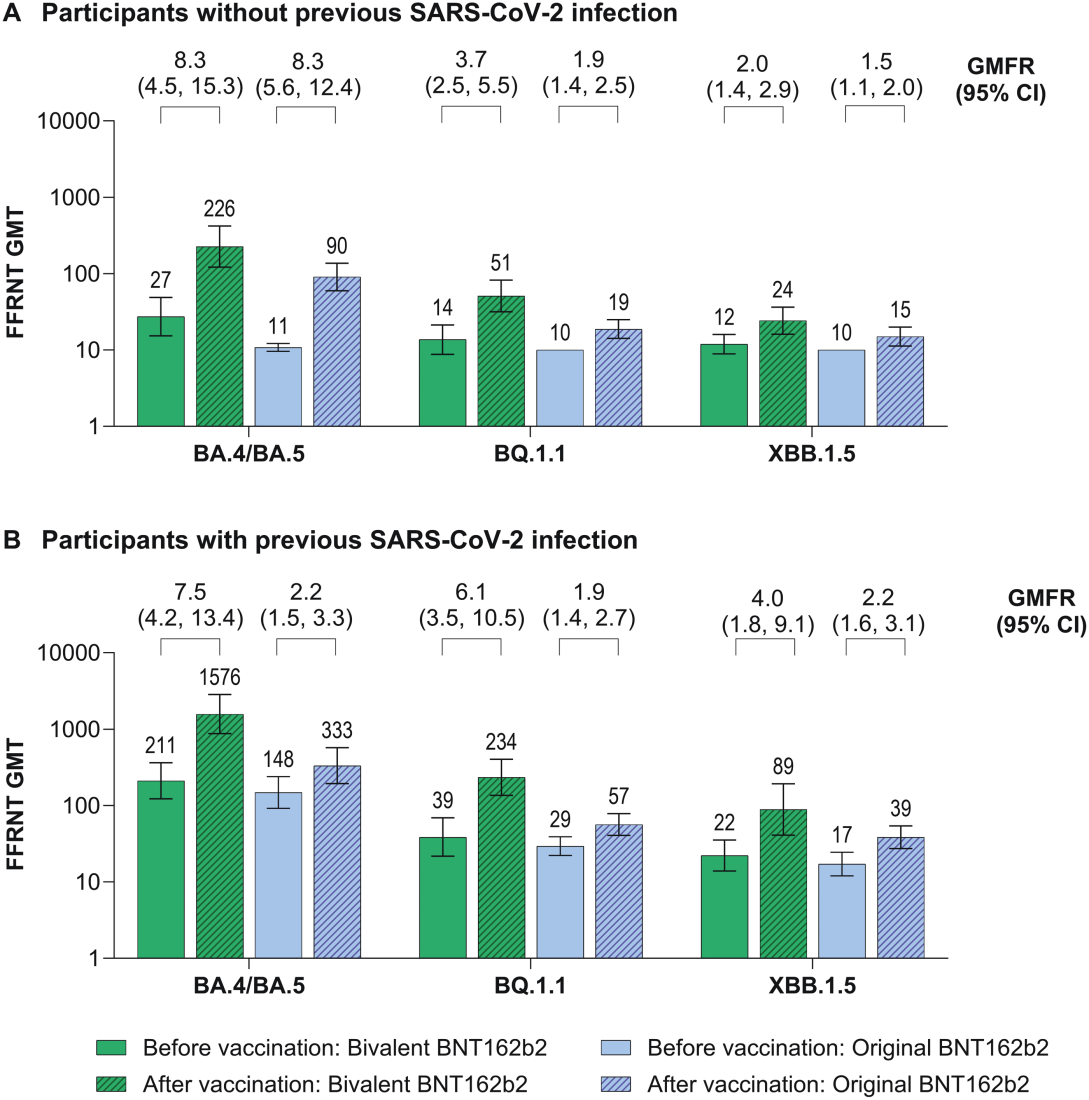
SARS-CoV-2 fluorescent focus reduction neutralization test assay results before and 1 month after vaccination with bivalent BNT162b2 (dose 4) or original BNT162b2 (dose 3) for Omicron BA.4/BA.5, BQ.1.1, and XBB.1.5 strains in children 6 months to <5 years old (A) without previous SARS-CoV-2 infection and (B) with previous SARS-CoV-2 infection. Shown are GMTs before and 1 month after vaccination and associated GMFRs. Data are for the evaluable SARS-CoV-2 variant immunogenicity subset and include participants with (*n* = 9–10) or without (*n* = 17–20) evidence of previous SARS-CoV-2 infection (Supplementary Table 2). This subset included 30 participants who had sufficient blood sample volume for additional testing among the first 24 and 36 participants assigned in the 6 months to <2 years age group and 2 years to <5 years age group, respectively, and had ≥1 valid and determinate immunogenicity result within the required window. GMTs are shown immediately above the bars and GMFRs and 95% CIs from before to 1 month after vaccination are shown in brackets above the bars. GMTs, GMFRs, and associated 95% CIs were calculated by exponentiating the mean logarithm of the titers (GMTs) or fold rises (GMFRs) and the corresponding CIs (based on the Student’s *t* distribution); assay results below the LLOQ were set to 0.5 × LLOQ. Data for original BNT162b2 are in participants from the original pediatric study (NCT04816643) who were matched by age, baseline SARS-CoV-2 status, and time since previous BNT162b2 dose (see Supplementary Table 5 for demographic details for these participants and those of the evaluable SARS-CoV-2 variant immunogenicity population of the current study). The definition of with or without previous SARS-CoV-2 infection is provided in the Supplementary Appendix. FFRNT, fluorescent focus reduction neutralization test; GMT, geometric mean titer; GMFR, geometric mean-fold rise.

In a subset of 6-month- to <5-year-olds, the neutralizing response against Omicron BQ.1.1 and XBB.1.5 was overall reduced compared with BA.4/BA.5 (Figure 2). However, responses against all Omicron sublineages tested were higher in the bivalent BNT162b2 group compared with the original BNT162b2 group.

### Safety

**Figure 3.**
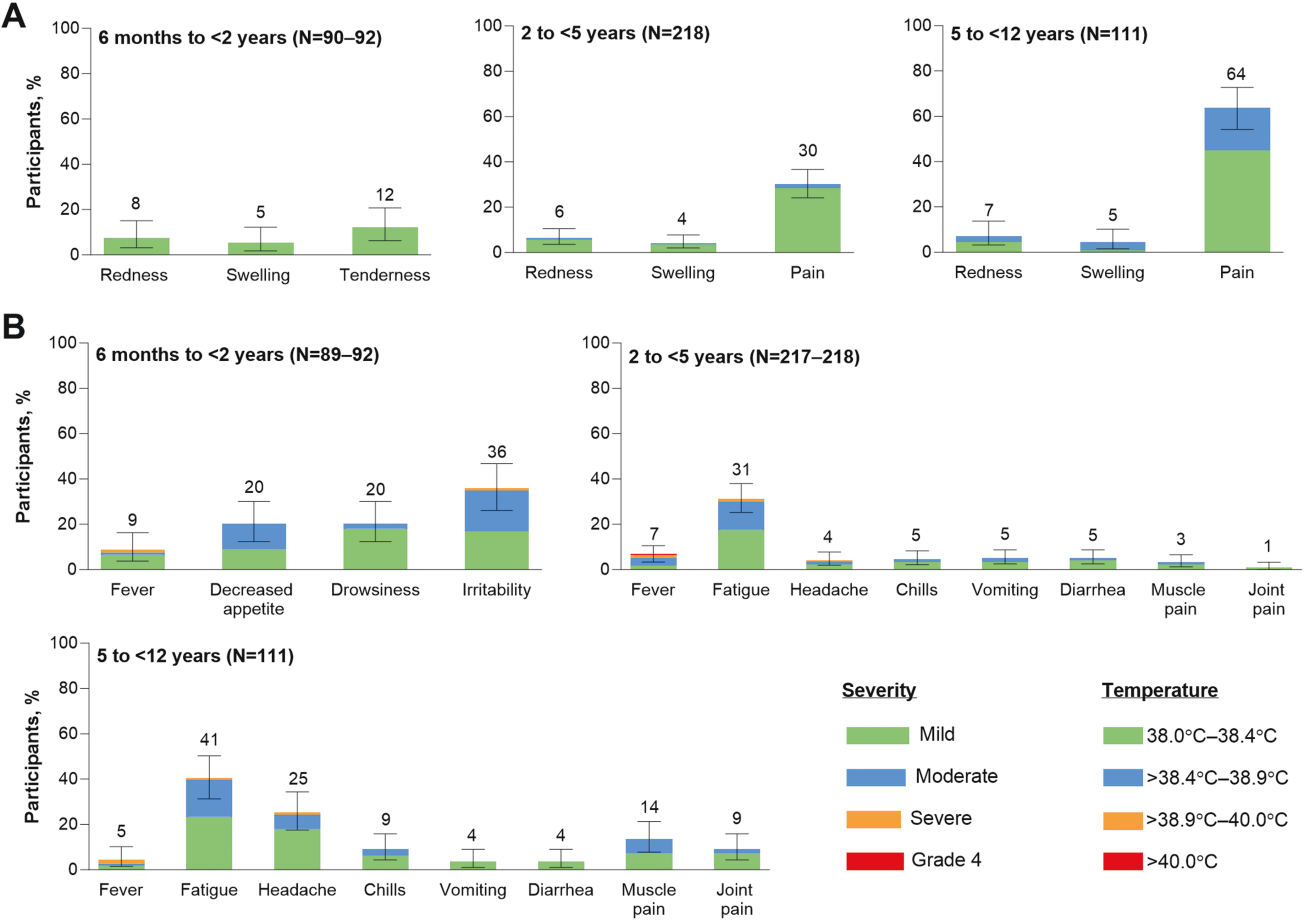
(A) Local reactions and (B) systemic events occurring within 7 days after receipt of dose 4 with bivalent BNT162b2. Data are for the safety population (Supplementary Table 2). Severity grading for each local reaction and systemic event is provided in Supplementary Table 1. Error bars represent 95% CIs and numbers above the error bars indicate the percentage of participants in each group reporting the specified local reaction or systemic event rounded to whole numbers.

Tenderness and injection-site pain were the most common local reactions in 6-month- to <2-year-olds and 2- to <12-year-olds, respectively (Figure 3A). The most common systemic event was irritability in 6-month- to <2-year-olds and fatigue in 2- to <12-year-olds (Figure 3B). Most reactogenicity events were mild to moderate. Severe reactogenicity events were infrequent, with no grade 4 (life-threatening) events. One 40.3°C fever was reported in a 4-year-old on Day 6 after vaccination, resolving within 2 days; headache, fatigue, chills, sneezing, and cough were also reported, with subsequent diagnosis of otitis media and pneumonia. The fever was considered unlikely to be vaccine-related by the investigator. Overall, the median onset was 1–2 days for local reactions and 2–6 days for systemic events. Median duration was 1–2 days for local reactions and systemic events.

The frequencies of AEs through 1 month and 6 months after vaccination were 10.9% and 13.0%, respectively, in 6-month- to <2-year-olds and 3.5%–4.6% and 6.2%–9.2%, respectively, in 2- to <12-year-olds (Supplementary Figure 3). A palpable left axillary lymph node (after left arm vaccination) was reported in one 5- to <12-year-old with onset 1 day after vaccination and was considered vaccine-related; the unsolicited event was of moderate severity and resolved within 3 days. No related AEs were severe, and most were consistent with reactogenicity events. One SAE was reported (breathing-related sleep disorder in a 2- to <5-year-old occurring approximately 2.5 months after vaccination that was classified as severe and not considered vaccine-related by the investigator). An additional severe AE was reported (influenza in a 5- to <12-year-old). No AEs leading to discontinuation were reported. No cases of anaphylaxis or hypersensitivity, appendicitis, Bell’s palsy, myocarditis, or pericarditis were reported.

### COVID-19 and MIS-C Surveillance

At data cutoff, there were 29 reports of COVID-19 in 6-month- to <5-year-olds (median 6.3 months of follow-up), and 7 reports in 5- to <12-year-olds (median 6.3 months of follow-up); the most common sublineage identified was XBB.1.5 in 11 participants overall (Supplementary Table 9). No cases were severe or resulted in hospitalization, and the median (range) time from the date of vaccination to the date of first COVID-19 symptom was 69 (6–130) days and 112 (45–173) days in 6-month- to <5-year-olds and 5- to <12-year-olds, respectively. There were no cases of MIS-C.

## DISCUSSION

In this analysis, bivalent BNT162b2 as a fourth dose elicited a robust immune response in infants and children 6 months to <12 years old, with a safety and tolerability profile immediately and in the month following vaccination similar to that seen with original BNT162b2 in infants, toddlers, and children [13, 15].

Among 6-month- to <5-year-old children, bivalent BNT162b2 given as a fourth dose met predefined immunogenicity superiority and noninferiority criteria when compared with a third dose of original BNT162b2. In a descriptive analysis without prespecified hypothesis testing, a similar trend in immune response was noted in 5- to <12-year-old children.

Among both SARS-CoV-2 baseline positive and negative 6-month- to <5-year-old participants, neutralizing titers against Omicron BA.4/BA.5 were higher in the bivalent BNT162b2 group compared with those in the original BNT162b2 group at prevaccination and more substantially at 1 month after vaccination. In a subset of 6-month- to <5-year-olds, postvaccination Omicron XBB.1.5 and BQ.1.1 neutralization titers were overall lower compared with Omicron BA.4/BA.5 titers. However, for all Omicron sublineages, titers were higher in the bivalent BNT162b2 group compared with the original BNT162b2 group.

The observed safety profile of bivalent BNT162b2 given as a fourth dose was consistent with the known safety profile of original BNT162b2 in infants, toddlers, and children, including real-world data [13, 15, 22]. Reactogenicity events were generally mild to moderate, severe reactogenicity events were infrequent, and none were life-threatening. No AEs led to discontinuation.

Limitations of this study include the short-term follow-up to assess the duration of immune responses. However, it was important to understand the immediate potential value of bivalent BNT162b2. Other limitations include that immunogenicity comparisons to original BNT162b2 were conducted with data from another clinical trial conducted at a different time and bivalent BNT162b2 given as a fourth dose was compared with original BNT162b2 given as a third dose. Despite the temporal distinction, most participants in the original BNT162b2 group received dose 3 in the spring of 2022 at a time of Omicron BA.1/BA.2 strain dominance. Matching of comparator groups for immunogenicity assessments did not include time since SARS-CoV-2 infection; as immune responses wane over time, this could have influenced comparability between groups. The population was also predominantly White and only from the United States.

Virus evolution is unpredictable [16], and future effects of SARS-CoV-2 evolution on human health will depend on the emergence rate of such antigenically distinct lineages, their virulence, and the ability of vaccine manufacturers to update vaccines [23]. Although the incidence of severe illness remains low relative to older adults, hospitalization rates among children were higher during the Omicron compared with the ancestral SARS-CoV-2 period [24]. Children therefore remain at risk due to poor uptake of additional vaccine doses [10], as well as antigenic divergence between predominant circulating variants and those in current vaccines.

Given the SARS-CoV-2 evolution to date [23], COVID-19 vaccines will likely need to continue to be updated periodically to maintain protection [25]. Although BA.4/BA.5-adapted bivalent BNT162b2 vaccine maintained early effectiveness in adults, including against XBB/XBB1.5 [26, 27], there were early signs of waning effectiveness after 2–4 months, including against severe disease [28–30]. A large real-world US study of >24 000 COVID-19 cases and >99 000 negative controls, conducted from August 2022 to April 2023, found that effectiveness of BA.4/BA.5-adapted bivalent BNT162b2 booster against outpatient visits waned from 35% at 0–3 months to 6% at 4–7 months, but that waning was not yet evident against severe disease outcomes at 4–7 months [31]. In mid-2023, the US Food and Drug Administration (FDA) and World Health Organization recommended an XBB monovalent variant vaccine for the 2023/2024 season that was a closer match to currently predominant strains [25, 32]. The FDA subsequently authorized an updated monovalent XBB.1.5-adapted BNT162b2 for use in individuals ≥6 months of age [33, 34].

In conclusion, these safety and immunogenicity data support a favorable benefit-risk profile for the administration of bivalent BNT162b2 as a fourth dose in 6-month- to <12-year-old children. The collective data support the use of COVID-19 strain-adapted vaccines in this pediatric age group.

## Supplementary Data

Supplementary materials are available at the *Journal of The Pediatric Infectious Diseases Society* online (http://jpids.oxfordjournals.org).

piae062_suppl_Supplementary_Tables_1-9_Figures_1-3

## Data Availability

Upon request, and subject to review, Pfizer will provide the data that support the findings of this study. Subject to certain criteria, conditions, and exceptions, Pfizer may also provide access to the related individual de-identified participant data. See https://www.pfizer.com/science/clinical-trials/data-and-results for more information.

## Notes

***Financial support.*** This study was sponsored by BioNTech and funded by Pfizer Inc. and BioNTech.

***Potential conflicts of interest:*** Lawrence D. Sher reports other (clinical investigator): Pfizer. Yvonne Maldonado reports other (site investigator): Pfizer (COVID-19 and RSV maternal vaccine clinical trials) and other (DSMB member): Pfizer non-COVID-19 vaccine trials, NIH ACTIV-2 trials. Emmanuel B. Walter reports other (clinical investigator): Pfizer, Moderna, Sequiris, Najit Technologies, Clinetic; advisor: Vaxcyte; and consultant: Iliad Biotechnologies. Flor M. Muñoz reports other (DSMB): Pfizer, Moderna (at the time of the study), Meissa Vaccines, Dynavax, NIH; consultant: Moderna, Sanofi, AstraZeneca, GSK, Merck; research support (to institution): Pfizer pediatric COVID-19 vaccines, Gilead remdesivir in children, NIH COVID-19 vaccines in pregnancy, CDC respiratory virus surveillance. Grant C. Paulsen reports research support (to institution): Pfizer and Moderna. Janet A. Englund reports consultant: AbbVie, AstraZeneca, Ark Biopharma, Meissa Vaccines, Moderna, Pfizer, Sanofi Pasteur; and research support: Pfizer, GlaxoSmithKline, AstraZeneca, Moderna, Merck. Kawsar R. Talaat reports research support (to institution): Pfizer, Sanofi; other (DSMB): Merck, Takeda, Intralytix, NIAID. Elizabeth D. Barnett reports research funds to her institution from Pfizer, consultant to Sobi, royalties from UpToDate and Elsevier, and honoraria from BMJ Best Practice and AAP. Satoshi Kamidani reports research support: Pfizer (funding provided to Emory University for a current trial), Emergent BioSolutions (funding provided to Emory University for a clinical trial), CDC (research grant provided to Emory University), NIH (grant provided to Emory University for vaccine trials). Shelly Senders reports none. Eric A.F. Simões reports research support: Bill and Melinda Gates Foundation, National Institutes of Health, USAID, AstraZeneca, Johnson & Johnson, Merck & Co Inc., Novavax, Pfizer, Regeneron, Roche; consultant: Bill and Melinda Gates Foundation, CDC, USAID, WHO, AbbVie Inc, Abbott Diagnostics, GSK plc, Johnson & Johnson, Pfizer; and other (travel): Bill and Melinda Gates Foundation, CDC, National Institutes of Health, USAID, WHO, AstraZeneca, Pfizer, Roche. Özlem Türeci reports BioNTech/Board membership and board member/Advisor/Consultant/Honoraria/Intellectual property/Patents/Other-Cofounder and Chief Medical Officer/Stock. Uğur Şahin reports BioNTech/Other-employee and board member/Stock. All other authors are employees of Pfizer Inc and may hold stock or stock options.
