# Supplementary material for: Bivalent Omicron BA.4/BA.5 BNT162b2 Vaccine in 6-Month- to <12-Year-Olds
Source: J Pediatric Infect Dis Soc. 2024 Jun 11;13(8):421–9. doi: 10.1093/jpids/piae062 (PMC11344470; doi:10.1093/jpids/piae062)

**Supplementary Appendix**

[List of Investigators 2](#_Toc161041161)

[Eligibility Criteria 5](#_Toc161041162)

[Prohibited Medications and Treatments During the Study 5](#_Toc161041163)

[Ethical Conduct 5](#_Toc161041164)

[Calculations of geometric mean titers (GMTs), geometric mean ratios (GMRs), geometric mean-fold rises (GMFRs), and seroresponse 6](#_Toc161041165)

[Baseline SARS-CoV-2 Status Definitions 6](#_Toc161041166)

[Procedures for Monitoring of Potential Myocarditis or Pericarditis 7](#_Toc161041167)

[Supplementary Table 1. Severity grading scale for local reactions and systemic events 8](#_Toc161041168)

[Supplementary Table 2. Study populations 10](#_Toc161041169)

[Supplementary Table 3. Demographic characteristics 11](#_Toc161041170)

[Supplementary Table 4. Demographic characteristics of evaluable immunogenicity population 5 to <12 years of age who received bivalent BNT162b2 in the current study and immunogenicity comparator group of participants 5 to <12 years of age who received original BNT162b2 in the original pediatric study 13](#_Toc161041171)

[Supplementary Table 5. Demographic characteristics of evaluable SARS-CoV-2 variant immunogenicity subset 6 months to <5 years of age who received bivalent BNT162b2 in the current study and immunogenicity comparator group of participants 6 months to <5 years of age who received original BNT162b2 in the original pediatric study 14](#_Toc161041172)

[Supplementary Table 6. Model-adjusted geometric mean ratio of SARS-CoV-2 neutralization assay titers: current study 6 months to <2 years of age and 2 to <5 years of age to comparator group of participants 6 months to <2 years of age and 2 to <5 years of age who received original BNT162b2 in the original pediatric study 15](#_Toc161041173)

[Supplementary Table 7. Model-adjusted geometric mean ratio of SARS-CoV-2 neutralization assay titers: current study 5 to <12 years of age to comparator group of participants 5 to <12 years of age who received original BNT162b2 in the original pediatric study 16](#_Toc161041174)

[Supplementary Table 8. Adjusted difference in percentages of participants with seroresponse 1 month after vaccination between bivalent BNT162b2 (dose 4) in the current study and the comparator group of participants 5 to <12 years of age who received original BNT162b2 (dose 3) in the original pediatric study 17](#_Toc161041175)

[Supplementary Table 9. SARS-CoV-2 variants for the first COVID-19 occurrence after study vaccination 18](#_Toc161041176)

[Supplementary Figure 1. Vaccine assignment and administration for participants (A) 6 months to <5 years and (B) 5 to <12 years of age 19](#_Toc161041177)

[Supplementary Figure 2. SARS-CoV-2 neutralization assay results before and 1 month after vaccination with bivalent BNT162b2 (dose 4) or original BNT162b2 (dose 3) for the (A) Omicron BA.4/BA.5 strain and (B) ancestral strain in children 6 months to <5 years old and 5 to <12 years old 20](#_Toc161041178)

[Supplementary Figure 3. Adverse events occurring through (A) 1 month and (B) 6 months after receipt of dose 4 with bivalent BNT162b2 22](#_Toc161041179)

#### List of Investigators

| **Principal Investigator** | **Sub-Investigator^a^** | **Institute** | **Location** |
| --- | --- | --- | --- |
| Acevedo, Armando | Deiros, Giselle  Canto, Alina Monteagudo  Navas, Mary  Pino, Bertha  Portela, Ana  Prieto, Christine  Rodriguez, Hector | Acevedo Clinical Research Associates | Miami, FL |
| Barnett, Elizabeth | Buncher, Noah  Campbell, Jeffrey  Cooper, Ellen  Fiore, Hannah  Moloney, Carole  Pelton, Stephen  Sabharwal, Vishakha | Boston Medical Center | Boston, MA |
| Englund, Janet | Korkowski, Sarah  Mohan, Kathleen  Ruedebusch, Paula  Vora, Surabhi  Waghmare, Alpana | Seattle Children’s Hospital | Seattle, WA |
| Faggett, Walter | Elliott, Tollie | Emerson Clinical Research Institute | Washington, DC |
| Hartman, Aaron | Decker, Raymond  Kaundal, Rishika  Lockard, Gretchen | Virginia Research Center | Midlothian, VA |
| Kamidani, Satoshi  (replaced Anderson, Evan) | Anderson, Larry  Anderson, Sakeena  Bower, Leisa  DiMaggio, Langdon  Dreyer, Alexandria  Hardison, Cindy  Kettle, Peggy  Lee, Kelly  Lewis, Marcia  Lubbers, Cindy  Macoy, Lisa  Nolan, Lauren  Peters, Etza  Rostad, Christina  Stephens, Kathleen | Emory Children’s Center, Emory University School of Medicine | Atlanta, GA |
| Li, Simon | Gaur, Sunanda  Louis, Cassandra  Ramagopal, Maya | Rutgers University  Rutgers Robert Wood Johnson Medical School | New Brunswick, NJ |
| Maldonado, Yvonne | Bollyky, Jennifer  Govindarajan, Prasanthi  Saxena, Jamie  Zhang, Hongqing | Clinical and Translational Research Unit & Spectrum Biobank | Palo Alto, CA |
| Meyer, Jay | Nutsch, Chelsie  West, Whitney | Velocity Clinical Research, Lincoln | Lincoln, NE |
| Muñoz, Flor | Bocchini, Claire  Mirani, Gayatri  Moulton, Elizabeth  Ruderfer, Daniel  Valencia Deray, Kristen | Texas Children’s Hospital | Houston, TX |
| Nayak, Jennifer | Caserta, Mary  Johnson, Jennifer | University of Rochester Medical Center | Rochester, NY |
| Paulsen, Grant | Brady, Rebecca  Buschle, Kristen  Dickey, Michelle  Frenck, Robert  Kidd, Jamie  Scaggs-Huang, Felicia  Spearman, Paul  Widdice, Eleanor | Cincinnati Children’s Hospital Medical Center | Cincinnati, OH |
| Senders, Shelly | Brennan, Molly  Fillioe, Caitlin  Jezerinac, Nicholas | Senders Pediatrics | South Euclid, OH |
| Sharp, Stephan | Caldwell, Michael | Clinical Research Associates | Nashville, TN |
| Sher, Lawrence | Palanjian, Alissa | Peninsula Research Associates | Rolling Hills Estates, CA |
| Simões, Eric | Feiten, Daniel  Kaleugher, Elizabeth | PediaClinic | Highlands Ranch, CO |
| Talaat, Kawsar | Atkinson, Rachel  Dhanjani, Kamal  Feijoo, Brittany  Na, Bin Yu | Center for Immunization Research Inpatient Unit, Johns Hopkins Bayview Medical Center | Baltimore, MD |
| Towner, William | Moss, Robert  Rosengart, Ronald | Kaiser Permanente | Los Angeles, CA |
| Walter, Emmanuel | Cassas, Christy  Moody, Michael  Smith, Michael | Duke Vaccine and Trials Unit | Durham, NC |
| Wisman, Paul | Armengol, Carlos  Jones, Amanda  Perriello, L. Paige | Pediatric Research of Charlottesville | Charlottesville, VA |

^a^ At any time during the study.

#### Eligibility Criteria

Individuals were excluded from the study if they were immunocompromised or had a suspected immunodeficiency, bleeding diathesis or condition associated with prolonged bleeding, that would contraindicate an intramuscular injection. Individuals were also excluded if there was any medical or laboratory abnormality that may increase the risk of study participation or make the participants inappropriate for the study. Individuals were excluded if they had a history of severe adverse reaction associated with a vaccine and/or severe allergic reaction (eg, anaphylaxis) to any component of study intervention. Individuals were also excluded if they had received a medication intended to prevent COVID-19 or had a previous or current diagnosis of MIS-C (multisystem inflammatory syndrome in children).

#### Prohibited Medications and Treatments During the Study

Besides as outlined in the exclusion criteria and in the main text, the following were not permitted:

- Receipt of systemic treatment with known immunosuppressant medication (including cytotoxic agents or systemic corticosteroids, eg, for cancer or an autoimmune disease) or radiotherapy, within 60 days before ennrollment through the conclusion of the study.
- Systemic corticosteroids (≥2 mg/kg of body weight or ≥20 mg/day of prednisone or equivalent for participants weighing >10 kg) for ≥14 days was prohibited from 28 days before enrollment through 28 days after administration of study intervention. Inhaled/nebulized, intra-articular, intrabursal, or topical (skin or eyes) corticosteroids were permitted.
- Receipt of blood/plasma products, immunoglobulin, or monoclonal antibodies (except palivizumab), from 60 days before study intervention administration, or receipt of any passive antibody therapy specific to COVID-19 from 90 days before study intervention administration, or planned receipt throughout the study.

#### Ethical Conduct

The study was conducted in accordance with ethical principles derived from international guidelines, including the Declaration of Helsinki and CIOMS International Ethical Guidelines, and applicable ICH GCP guidelines, laws, and regulations, including privacy laws. Parents or legal guardians provided written informed consent and participants provided assent when capable.

#### Calculations of geometric mean titers (GMTs), geometric mean ratios (GMRs), geometric mean-fold rises (GMFRs), and seroresponse

GMTs, GMFRs and GMRs

GMTs and GMFRs with two-sided 95% CIs were calculated by exponentiating the mean logarithm of the titers and fold rises, respectively, and the corresponding CIs (based on Student’s *t* distribution). Assay results below the lower limit of quantitation (LLOQ) were set to 0.5 × LLOQ.

Model-adjusted GMRs and associated 95% CIs were calculated by exponentiating the difference in least squares (LS) means and the corresponding CIs based on analysis of logarithmically transformed assay results using a linear regression model that included the baseline neutralizing titer, postbaseline infection status, age subgroup (for 6 months to <5 years), and vaccine group as covariates.

Seroresponse

Seroresponse was defined as achieving a ≥4-fold rise from baseline (before the first study vaccination [fourth dose]). If the baseline measurement was below the LLOQ, the postvaccination measure of ≥4 × LLOQ was considered seroresponse. For the comparator group of participants from the original pediatric study (Study C4591007 [NCT04816643]), seroresponse was defined as achieving a ≥4-fold rise from before the third dose. If the pre–third dose measurement was below the LLOQ, the postvaccination measure of ≥4 × LLOQ was considered seroresponse. The exact two-sided 95% CIs for percentages of participants with seroresponse was calculated using the Clopper-Pearson method.

The adjusted difference in seroresponse rate between the two vaccine groups, and associated 95% CIs, were based on the Miettinen and Nurminen method stratified by baseline neutralizing titer category (<median or ≥median). The median of baseline neutralizing titers was calculated based on the pooled data in the two comparator groups.

#### Baseline SARS-CoV-2 Status Definitions

For participants who received bivalent BNT162b2 in current study, baseline refers to before the study vaccination (dose 4 with bivalent BNT162b2). SARS-CoV-2 positive at baseline was defined as having a positive N-binding antibody result or positive nucleic acid amplification test (NAAT) result at the study visit before receipt of bivalent BNT162b2, or a medical history of COVID-19. SARS-CoV-2 negative at baseline was defined as having a negative N-binding antibody and NAAT result at the study visit before receipt of bivalent BNT162b2, and no medical history of COVID-19.

For the comparative group participants who received three doses of original BNT162b2 in the original pediatric study, baseline refers to before dose 3 of original BNT162b2. SARS-CoV-2 positive at baseline was defined as a positive N-binding antibody result at the dose 1, 1 month after dose 2 (if available), or dose 3 visit; positive NAAT result at the dose 1, dose 2, dose 3 visit, or any unscheduled illness visit up to the dose 3 visit; or a medical history of COVID-19. SARS-CoV-2 negative at baseline was defined as a negative N-binding antibody result at the dose 1, 1 month after dose 2 (if available), and dose 3 visit; negative NAAT result at the dose 1, dose 2, dose 3, and any unscheduled visit up to the dose 3 visit; and no medical history of COVID-19.

#### Procedures for Monitoring of Potential Myocarditis or Pericarditis

Any study participant who reported acute chest pain, shortness of breath, palpitations, or any other symptom(s) that might be indicative of myocarditis or pericarditis within 4 weeks after a study vaccination was to be specifically evaluated, preferably by a cardiologist, for possible myocarditis or pericarditis. In addition to a clinical evaluation, an electrocardiogram (ECG) and measurement of troponin levels were to be performed. If myocarditis or pericarditis was suspected based upon the initial evaluation, a cardiac echocardiogram and/or cardiac magnetic resonance study were performed.

#### Supplementary Table 1. Severity grading scale for local reactions and systemic events

|  | **Mild** | **Moderate** | **Severe** |
| --- | --- | --- | --- |
| **Local reaction** |  |  |  |
| **<2 years of age** |  |  |  |
| Tenderness | Hurts if gently touched  (eg, whimpers, winces, protests, or withdraws) | Hurts if gently touched with crying | Causes limitation of limb movement |
| Redness | 1–4 caliper units (0.5–2.0 cm) | 5–14 caliper units (>2.0–7.0 cm) | >14 caliper units (>7 cm) |
| Swelling | 1–4 caliper units (0.5–2.0 cm) | 5–14 caliper units (>2.0–7.0 cm) | >14 caliper units (>7 cm) |
| **2–<12 years of age** |  |  |  |
| Pain | Does not interfere with activity | Interferes with activity | Prevents daily activity |
| Redness | 1–4 caliper units (0.5–2.0 cm) | 5–14 caliper units (>2.0–7.0 cm) | >14 caliper units (>7 cm) |
| Swelling | 1–4 caliper units (0.5–2.0 cm) | 5–14 caliper units (>2.0–7.0 cm) | >14 caliper units (>7 cm) |
| **Systemic event** |  |  |  |
| **<2 years of age** |  |  |  |
| Decreased appetite (loss of appetite) | Decreased interest in eating | Decreased oral intake | Refusal to eat |
| Drowsiness (increased sleep) | Increased or prolonged sleep bouts | Slightly subdued interfering with daily activity | Disabling; not interested in usual daily activity |
| Irritability (fussiness)^a^ | Easily consolable | Requiring increased attention | Inconsolable; crying cannot be comforted |
| **2–<12 years of age** |  |  |  |
| Vomiting | 1–2 times in 24 hours | >2 times in 24 hours | Requires IV hydration |
| Diarrhea | 2–3 loose stools/24 hours | 4–5 loose stools/24 hours | ≥6 loose stools/24 hours |
| Headache | Does not interfere with activity | Some interference with activity | Prevents daily routine activity |
| Fatigue/tiredness | Does not interfere with activity | Some interference with activity | Prevents daily routine activity |
| Chills | Does not interfere with activity | Some interference with activity | Prevents daily routine activity |
| Muscle pain | Does not interfere with activity | Some interference with activity | Prevents daily routine activity |
| Joint pain | Does not interfere with activity | Some interference with activity | Prevents daily routine activity |

IV=intravenous.

For the fever scale, refer to the legend and scale of **Figure 3**.
^a^Synonymous with restless sleep or decreased sleep.

#### Supplementary Table 2. Study populations

| **Population** | **Definition** | **N** | **Notes** |
| --- | --- | --- | --- |
| Safety | All participants who received dose 4 with bivalent BNT162b2 | 6 months–<2 years of age (N=92)  2–<5 years of age (N=218)  5–<12 years of age (N=113) | The population included in Supplementary Table 3; Figure 3; Supplementary Figure 3 |
| Evaluable immunogenicity (full group) | Participants who had ≥1 valid and determinate immunogenicity result within 28–42 days after dose 4 with bivalent BNT162b2 and had no important protocol deviations as determined by the clinician | 6 months–<2 years of age (N=78)  2–<5 years of age (N=196)  5–<12 years of age (N=113) | The population included in Supplementary Figure 2; Table 1; Supplementary Table 6; Supplementary Table 7; Supplementary Table 8 |
| Evaluable immunogenicity (per protocol) for hypothesis testing | Random sample from the evaluable immunogenicity population (full group) and comprising the same percentages of participants in each age group and baseline SARS-CoV-2 infection status group as the full group. | 6 months–<5 years of age (N=240) | The population included in Figure 1 |
| Evaluable SARS-CoV-2 variant immunogenicity subset | Participants who had ≥1 valid and determinate SARS-CoV-2 variant immunogenicity result within 28–42 days after dose 4 with bivalent BNT162b2 and had no important protocol deviations as determined by the clinician. | 6 months–<5 years of age (N=30) | The population included in Figure 2 |

####

#### Supplementary Table 3. Demographic characteristics

| **Characteristic** | **6 months–<2 years of age (N=92)** | **2–<5 years of age (N=218)** | **5–<12 years of age (N=113)** |
| --- | --- | --- | --- |
| Sex, n (%) |  |  |  |
| Male | 51 (55.4) | 105 (48.2) | 57 (50.4) |
| Female | 41 (44.6) | 113 (51.8) | 56 (49.6) |
| Race or ethnicity, n (%) |  |  |  |
| White | 64 (69.6) | 153 (70.2) | 66 (58.4) |
| Black | 2 (2.2) | 7 (3.2) | 9 (8.0) |
| Asian | 11 (12.0) | 18 (8.3) | 13 (11.5) |
| Multiracial | 15 (16.3) | 39 (17.9) | 22 (19.5) |
| Not reported | 0 | 1 (0.5) | 3 (2.7) |
| Hispanic/Latino | 18 (19.6) | 34 (15.6) | 23 (20.4) |
| Age at study vaccination |  |  |  |
| Mean (standard deviation) | 19.2 (3.37) months | 2.9 (0.84) years | 8.6 (1.65) years |
| Median (range) | 20 (11–23) months | 3 (2–4) years | 9.0 (5–11) years |
| Time since previous dose of original BNT162b2 |  |  |  |
| Median time (range), months | 5.3 (2.1–8.6) | 7.0 (2.1–8.6) | 5.5 (2.6–8.5) |
| <3 months, n (%) | 10 (10.9) | 12 (5.5) | 1 (0.9) |
| 3–<4 months, n (%) | 10 (10.9) | 13 (6.0) | 7 (6.2) |
| 4–<5 months, n (%) | 18 (19.6) | 19 (8.7) | 29 (25.7) |
| 5–<6 months, n (%) | 15 (16.3) | 27 (12.4) | 24 (21.2) |
| 6–<7 months, n (%) | 17 (18.5) | 37 (17.0) | 13 (11.5) |
| 7–<8 months, n (%) | 8 (8.7) | 61 (28.0) | 27 (23.9) |
| 8–<9 months, n (%) | 14 (15.2) | 49 (22.5) | 12 (10.6) |
| Baseline SARS-CoV-2 status,^a^ n (%) |  |  |  |
| Positive | 44 (47.8) | 81 (37.2) | 66 (58.4) |
| Negative | 42 (45.7) | 134 (61.5) | 47 (41.6) |
| Missing | 6 (6.5) | 3 (1.4) | 0 |
| Comorbidities (total),^b^ n (%) | 6 (6.5) | 24 (11.0) | 31 (27.4) |
| Comorbidities occurring in ≥1% in any group |  |  |  |
| Asthma | 0 | 2 (0.9) | 7 (6.2) |
| Attention deficit hyperactivity disorder | Not applicable | Not applicable | 14 (12.4) |
| Bronchial hyperreactivity | 2 (2.2) | 3 (1.4) | 0 |
| Congenital coronary artery malformation | 1 (1.1) | 0 | 0 |
| Premature baby | 3 (3.3) | 2 (0.9) | Not applicable |
| Obesity | Not applicable | 14 (6.4) | 10 (8.8) |

BMI=body mass index; NAAT=nucleic acid amplification test; N-binding=SARS-CoV-2 nucleoprotein–binding.

Results are for the safety population (defined in **Supplementary Table 1)**. Demographic characteristics for the evaluable immunogenicity populations and the immunogenicity comparator groups are provided in **Table 1** and **Supplementary Tables 4–5**.
^a^ According to N-binding antibody or NAAT result at the study vaccination visit (ie, dose 4 with bivalent BNT162b2), and medical history of COVID-19.
^b^ Number of participants who had ≥1 comorbidity that increases the risk of severe COVID-19: defined as participants who had ≥1 of the prespecified comorbidities as defined by Kim et al [34] (6 months–<12 years of age), and the Centers for Disease Control and Prevention [35] (6 months–<5 years of age), and/or obesity (BMI ≥95th percentile; 2–<12 years of age). Comorbidities were assessed at the first study visit for both studies.

#### Supplementary Table 4. Demographic characteristics of evaluable immunogenicity population 5 to <12 years of age who received bivalent BNT162b2 in the current study and immunogenicity comparator group of participants 5 to <12 years of age who received original BNT162b2 in the original pediatric study

| **Characteristic** | **Received bivalent BNT162b2 in current study** | **Received original BNT162b2 in original pediatric study** |
| --- | --- | --- |
|  | **Evaluable immunogenicity population (N=103)** | **Immunogenicity comparator group**^a^ **(N=113)** |
| Sex, n (%) |  |  |
| Male | 49 (47.6) | 63 (55.8) |
| Female | 54 (52.4) | 50 (44.2) |
| Race or ethnicity, n (%) |  |  |
| White | 63 (61.2) | 91 (80.5) |
| Black | 8 (7.8) | 4 (3.5) |
| Asian | 12 (11.7) | 11 (9.7) |
| Multiracial | 17 (16.5) | 4 (3.5) |
| Native Hawaiian or other Pacific Islander | 0 | 2 (1.8) |
| Not reported | 3 (2.9) | 1 (0.9) |
| Hispanic/Latino | 23 (22.3) | 16 (14.2) |
| Age at study vaccination, years |  |  |
| Mean (standard deviation) | 8.6 (1.65) | 8.6 (1.65) |
| Median (range) | 9.0 (5–11) | 9.0 (5–11) |
| Median time (range) since previous dose of original BNT162b2, months | 5.5 (3.5–8.5) | 6.5 (6.3–7.6) |
| Baseline SARS-CoV-2 status,^b^ n (%) |  |  |
| Positive | 59 (57.3) | 66 (58.4) |
| Negative | 44 (42.7) | 47 (41.6) |
| Comorbidities,^c^ n (%) | 28 (27.2) | 33 (29.2) |

BMI=body mass index; NAAT=nucleic acid amplification test; N-binding=SARS-CoV-2 nucleoprotein–binding.

^a^ Participants in the original pediatric study (NCT04816643) who were 5−<12 years old and who had received three doses of original BNT162b2 10 μg.
^b^ According to N-binding antibody or NAAT result at the study vaccination visit (ie, dose 4 with bivalent BNT162b2 for study participants), and medical history of COVID-19. See the **Supplementary Appendix** for the definitions for the immunogenicity comparator group.
^c^ Includes those who had ≥1 comorbidity that increases the risk of severe COVID-19: defined as participants who had ≥1 of the prespecified comorbidities as defined by Kim et al [34], and /or obesity (BMI ≥95th percentile).

#### Supplementary Table 5. Demographic characteristics of evaluable SARS-CoV-2 variant immunogenicity subset 6 months to <5 years of age who received bivalent BNT162b2 in the current study and immunogenicity comparator group of participants 6 months to <5 years of age who received original BNT162b2 in the original pediatric study

| **Characteristic** | **Received bivalent BNT162b2 in current study** | **Received original BNT162b2 in original pediatric study** |
| --- | --- | --- |
|  | **Evaluable immunogenicity population (N=30)** | **Immunogenicity comparator group**^a^ **(N=27)** |
| Sex, n (%) |  |  |
| Male | 13 (43.3) | 15 (55.6) |
| Female | 17 (56.7) | 12 (44.4) |
| Race or ethnicity, n (%) |  |  |
| White | 20 (66.7) | 23 (85.2) |
| Black | 2 (6.7) | 1 (3.7) |
| Asian | 2 (6.7) | 2 (7.4) |
| Multiracial | 6 (20.0) | 1 (3.7) |
| Hispanic/Latino | 11 (36.7) | 6 (22.2) |
| Age at study vaccination, months |  |  |
| Mean (standard deviation) | 32.0 (12.51) | 31.5 (13.06) |
| Median (range) | 30.5 (12, 57) | 29.0 (13, 58) |
| Median time (range) since previous dose of original BNT162b2, months | 7.0 (2.1, 8.6) | 7.0 (2.2, 9.1) |
| Baseline SARS-CoV-2 status,^b^ n (%) |  |  |
| Positive | 10 (33.3) | 9 (33.3) |
| Negative | 20 (66.7) | 17 (63.0) |
| Missing | 0 | 1 (3.7) |
| Comorbidities,^c^ n (%) | 4 (13.3) | 0 |

BMI=body mass index; NAAT=nucleic acid amplification test; N-binding=SARS-CoV-2 nucleoprotein–binding.

^a^ Participants in the original pediatric study (NCT04816643) who were 6 months−<5 years old and who had received three doses of original BNT162b2 3 μg.
^b^ According to N-binding antibody or NAAT result at the study vaccination visit (ie, dose 4 with bivalent BNT162b2 for study participants), and medical history of COVID-19. See the **Supplementary Appendix** for the definitions for the immunogenicity comparator group.
^c^ Includes those who had ≥1 comorbidity that increases the risk of severe COVID-19: defined as participants who had ≥1 of the prespecified comorbidities as defined by Kim et al [34], and the Centers for Disease Control and Prevention [35], and /or obesity (BMI ≥95th percentile for participants ≥2 years of age).

#### Supplementary Table 6. Model-adjusted geometric mean ratio of SARS-CoV-2 neutralization assay titers: current study 6 months to <2 years of age and 2 to <5 years of age to comparator group of participants 6 months to <2 years of age and 2 to <5 years of age who received original BNT162b2 in the original pediatric study

| **Characteristic** | **Received bivalent BNT162b2 in current study** | | **Received original BNT162b2 in original pediatric study** | |  |
| --- | --- | --- | --- | --- | --- |
|  | **n** | **Unadjusted**  **GMT**  **(95% CI)** | **n** | **Unadjusted**  **GMT**  **(95% CI)** | **Model-adjusted**  **GMR**  **(95% CI)** |
| **6 months to <2 years of age** |  |  |  |  |  |
| SARS-CoV-2 neutralization assay: Omicron BA.4/BA.5 NT50 | 63 | 1926.3  (1300.3, 2853.6) | 72 | 924.1 (702.5, 1215.6) | 1.61  (1.20, 2.18) |
| SARS-CoV-2 neutralization assay: ancestral strain NT50 | 63 | 6412.6 (5095.7, 8069.9) | 72 | 6719.0 (5502.4, 8204.6) | 0.84  (0.65, 1.08) |
| **2 to <5 years of age** |  |  |  |  |  |
| SARS-CoV-2 neutralization assay: Omicron BA.4/BA.5 NT50 | 161 | 2359.1 (1896.8, 2934.1) | 167 | 739.7 (617.8, 885.6) | 2.13 (1.73, 2.62) |
| SARS-CoV-2 neutralization assay: ancestral strain NT50 | 161 | 7958.7 (6892.6, 9189.6) | 166 | 6449.5 (5670.3, 7335.9) | 0.94 (0.79, 1.11) |

GMR=geometric mean ratio; GMT=geometric mean titer; NT50=50% neutralizing titer.

Data are for the evaluable immunogenicity population and include participants with or without evidence of previous SARS-CoV-2 infection (**Supplementary Table 2**). GMTs and two-sided CIs were calculated by exponentiating the mean logarithm of the titers and the corresponding CIs (based on the Student’s *t* distribution). Model-adjusted GMR and two-sided CIs were calculated by exponentiating the difference of the least squares means for the assay and the corresponding CIs based on analysis of the log-transformed assay results using a linear regression model with baseline log-transformed titers, post baseline infection status, age group, and vaccine group as covariates.

#### Supplementary Table 7. Model-adjusted geometric mean ratio of SARS-CoV-2 neutralization assay titers: current study 5 to <12 years of age to comparator group of participants 5 to <12 years of age who received original BNT162b2 in the original pediatric study

| **Characteristic** | **Received bivalent BNT162b2 in current study** | | **Received original BNT162b2 in original pediatric study** | |  |
| --- | --- | --- | --- | --- | --- |
|  | **n** | **Unadjusted**  **GMT**  **(95% CI)** | **n** | **Unadjusted**  **GMT**  **(95% CI)** | **Model-adjusted**  **GMR**  **(95% CI)** |
| SARS-CoV-2 neutralization assay: Omicron BA.4/BA.5 NT50 | 102 | 2189.9  (1742.8, 2751.7) | 113 | 1393.6  (1175.8, 1651.7) | 1.12  (0.92, 1.37) |
| SARS-CoV-2 neutralization assay: ancestral strain NT50 | 102 | 8245.9  (7108.9, 9564.9) | 113 | 7235.1  (6331.5, 8267.8) | – |

GMR=geometric mean ratio; GMT=geometric mean titer; NT50=50% neutralizing titer.

Data are for the evaluable immunogenicity population and include participants with or without evidence of previous SARS-CoV-2 infection (**Supplementary Table 2**). GMTs and two-sided CIs were calculated by exponentiating the mean logarithm of the titers and the corresponding CIs (based on the Student’s *t* distribution). Model-adjusted GMR and two-sided CIs were calculated by exponentiating the difference of the least squares means for the assay and the corresponding CIs based on analysis of the log-transformed assay results using a linear regression model with baseline log-transformed titers, post baseline infection status, and vaccine group as covariates.

#### Supplementary Table 8. Adjusted difference in percentages of participants with seroresponse 1 month after vaccination between bivalent BNT162b2 (dose 4) in the current study and the comparator group of participants 5 to <12 years of age who received original BNT162b2 (dose 3) in the original pediatric study

| **Characteristic** | **Received bivalent BNT162b2 (dose 4) in current study**  **(N=101)^a^** | | **Received original BNT162b2 (dose 3) in original pediatric study**  **(N=112)^a^** | | **Adjusted difference^d^ (95% CI)^e^** |
| --- | --- | --- | --- | --- | --- |
|  | **Unadjusted seroresponse** | | **Unadjusted seroresponse** | |  |
|  | **n^b^ (%)** | **(95% CI)^c^** | **n^b^ (%)** | **(95% CI)^c^** |  |
| SARS-CoV-2 neutralization assay: Omicron BA.4/BA.5 NT50 | 54 (53.5) | (43.3, 63.5) | 59 (52.7) | (43.0, 62.2) | 8.76 (–2.47, 19.99) |
| SARS-CoV-2 neutralization assay: ancestral strain NT50 | 31 (30.7) | (21.9, 40.7) | 62 (54.9) | (45.2, 64.2) | – |

GMR=geometric mean ratio; GMT=geometric mean titer; NT50=50% neutralizing titer.

Data are for the evaluable immunogenicity population and include participants with or without evidence of previous SARS-CoV-2 infection (**Supplementary Table 2**). Seroresponse was defined as achieving a ≥4-fold rise from baseline (before dose 4 for the current study and before dose 3 for the comparator group); if the baseline measurement was below the lower limit of quantitation (LLOQ), a postvaccination assay result ≥4 × LLOQ was considered a seroresponse.

^a^ Number of participants with valid and determinate assay results for the specified assay both before the relevant dose and at the given dose/sampling time point. These values are the denominators for the percentage calculations.

^b^ Number of participants with seroresponse for the given assay at the given dose/sampling time point.

^c^ Exact two-sided CI based on the Clopper and Pearson method.

^d^ Adjusted difference in proportions based on the Miettinen and Nurminen method stratified by baseline neutralizing titer category (< median, ≥ median), expressed as a percentage (bivalent BNT162b2 10 μg – BNT162b2 10 μg). The median of baseline neutralizing titers was calculated based on the pooled data in two comparator groups.

^e^ Two-sided CI, based on the Miettinen and Nurminen method for the difference in proportions stratified by baseline neutralizing titer category (< median, ≥ median), expressed as a percentage.

#### Supplementary Table 9. SARS-CoV-2 variants for the first COVID-19 occurrence after study vaccination

| **SARS-CoV-2 lineage^a^** | **Received bivalent BNT162b2 in current study** | | |  |
| --- | --- | --- | --- | --- |
|  | **6 months to  <2 years**  **(N=11)** | **2 to <5 years**  **(N=18)** | **6 months to  <5 years**  **(N=29)** | **5 to <12 years**  **(N=7)** |
| Overall | 11 (100.0) | 18 (100.0) | 29 (100.0) | 7 (100.0) |
| Omicron | 9 (81.8) | 13 (72.2) | 22 (75.9) | 7 (100.0) |
| Omicron BA.5.1.3 | 1 (9.1) | 0 | 1 (3.4) | 0 |
| Omicron BL.1 | 0 | 1 (5.6) | 1 (3.4) | 0 |
| Omicron BQ.1 | 0 | 2 (11.1) | 2 (6.9) | 1 (14.3) |
| Omicron BQ.1.1 | 0 | 2 (11.1) | 2 (6.9) | 0 |
| Omicron BQ.1.1.18 | 1 (9.1) | 0 | 1 (3.4) | 0 |
| Omicron BQ.1.1.35 | 1 (9.1) | 0 | 1 (3.4) | 0 |
| Omicron BQ.1.1.4 | 0 | 0 | 0 | 1 (14.3) |
| Omicron BQ.1.25 | 1 (9.1) | 0 | 1 (3.4) | 0 |
| Omicron BQ.1.5.35 | 0 | 0 | 0 | 1 (14.3) |
| Omicron CQ.1.1 | 1 (9.1) | 0 | 1 (3.4) | 0 |
| Omicron FD.2 | 0 | 1 (5.6) | 1 (3.4) | 0 |
| Omicron XBB.1.16.20 | 0 | 1 (5.6) | 1 (3.4) | 0 |
| Omicron XBB.1.5 | 3 (27.3) | 4 (22.2) | 7 (24.1) | 4 (57.1) |
| Omicron XBB.1.5.1 | 0 | 1 (5.6) | 1 (3.4) | 0 |
| Omicron XBB.1.5.14 | 0 | 1 (5.6) | 1 (3.4) | 0 |
| Omicron XBB.1.5.34 | 1 (9.1) | 0 | 1 (3.4) | 0 |
| Unknown^b^ | 2 (18.2) | 5 (27.8) | 7 (24.1) | 0 |

WHO=World Health Organization; PANGO=Phylogenetic Assignment of Named Global Outbreak.

Median follow-up was 6.3 months in both 6-month−<5-year-olds and 5−<12-year-olds.

^a^ Based on WHO Classification (Tracking SARS-CoV-2 variants [who.int]) and PANGO lineages (cov-lineages.org) and includes all descendent lineages.

^b^ Includes indeterminate results and nonquantifiable (QNS) or not sequenced samples.

#### Supplementary Figure 1. Vaccine assignment and administration for participants (A) 6 months to <5 years and (B) 5 to <12 years of age

At data cutoff (March 03, 2023 [panel A] and April 20, 2023 [panel B]), one participant from the 6 months to <5 years of age group had not yet completed the 1 month postvaccination study visit.


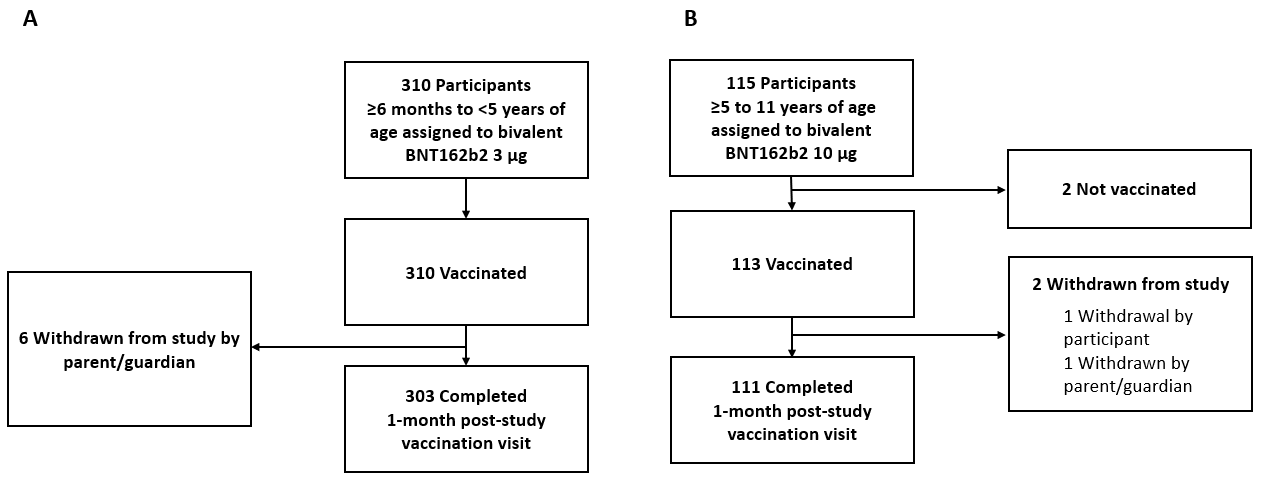


#### Supplementary Figure 2. SARS-CoV-2 neutralization assay results before and 1 month after vaccination with bivalent BNT162b2 (dose 4) or original BNT162b2 (dose 3) for the (A) Omicron BA.4/BA.5 strain and (B) ancestral strain in children 6 months to <5 years old and 5 to <12 years old

Shown are the GMTs before and 1 month after vaccination and associated GMFRs. Data are for the evaluable immunogenicity population and include participants with or without evidence of previous SARS-CoV-2 infection (**Supplementary Table 2**). GMTs are shown immediately above the bars and GMFRs from before to 1 month after vaccination are shown in brackets above the bars. GMTs, GMFRs, and associated 95% CIs were calculated by exponentiating the mean logarithm of the titers (GMTs) or fold rises (GMFRs) and the corresponding CIs (based on the Student’s t distribution); assay results below the LLOQ were set to 0.5 × LLOQ. Data for original BNT162b2 are in participants from the original pediatric study (NCT04816643) who were matched by age, and baseline SARS-CoV-2 positivity (6 months to <12 years old), and time since previous BNT162b2 dose (for 6 months to <5 years old only; see **Supplementary Tables 3–4** for demographic details for these participants and those of the evaluable immunogenicity population of the current study). Definition of SARS-CoV-2 positive or negative is provided in the **Supplementary Appendix**. GMT=geometric mean titer; GMFR=geometric mean-fold rise; LLOQ=lower limit of quantitation.


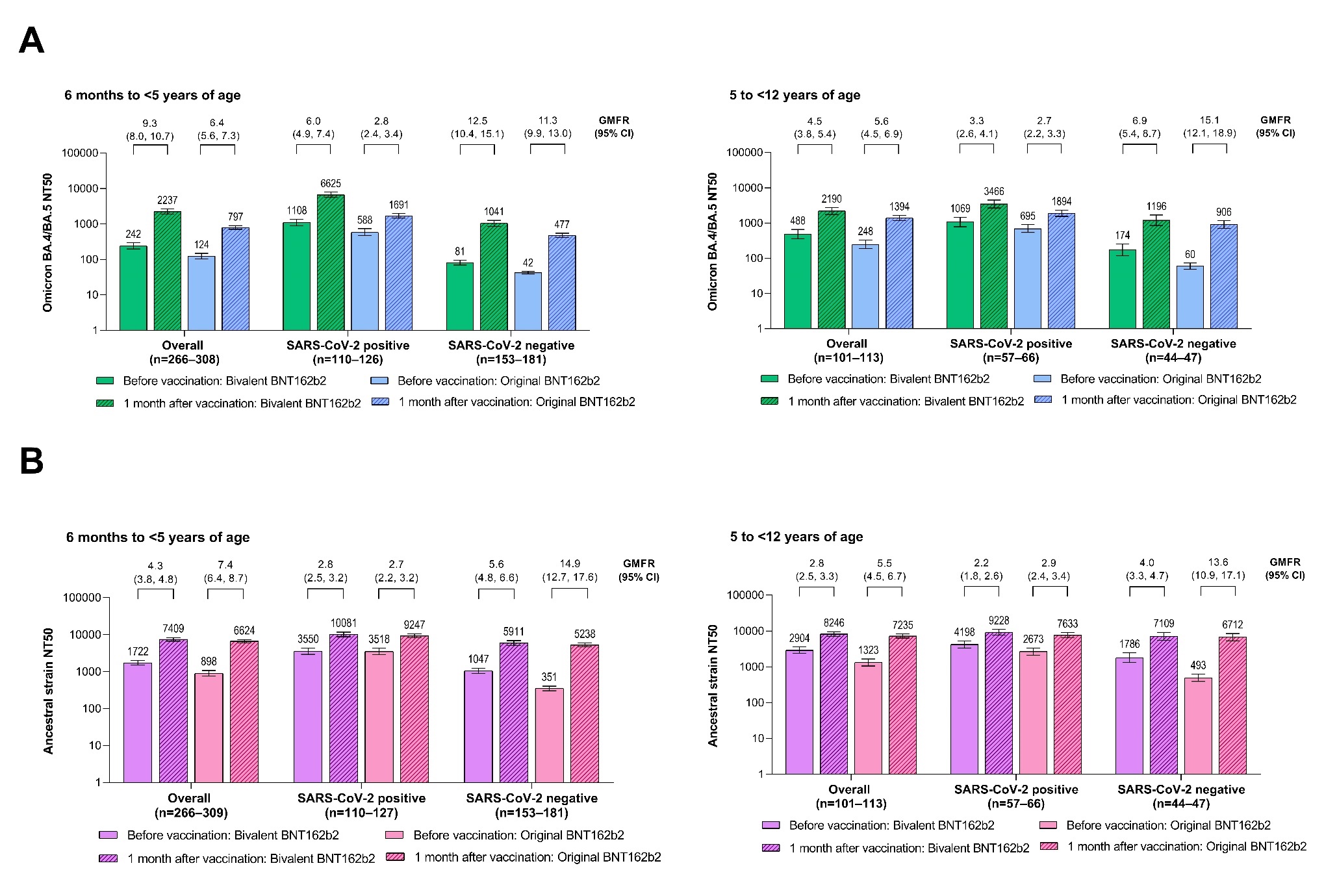


#### Supplementary Figure 3. Adverse events occurring through (A) 1 month and (B) 6 months after receipt of dose 4 with bivalent BNT162b2

Data are for the safety population (defined in **Supplementary Table 2**). The numbers above the bars show the percentage of participants who experienced ≥1 of the specified type of event. Related AEs were as assessed by the investigator. AE=adverse event.


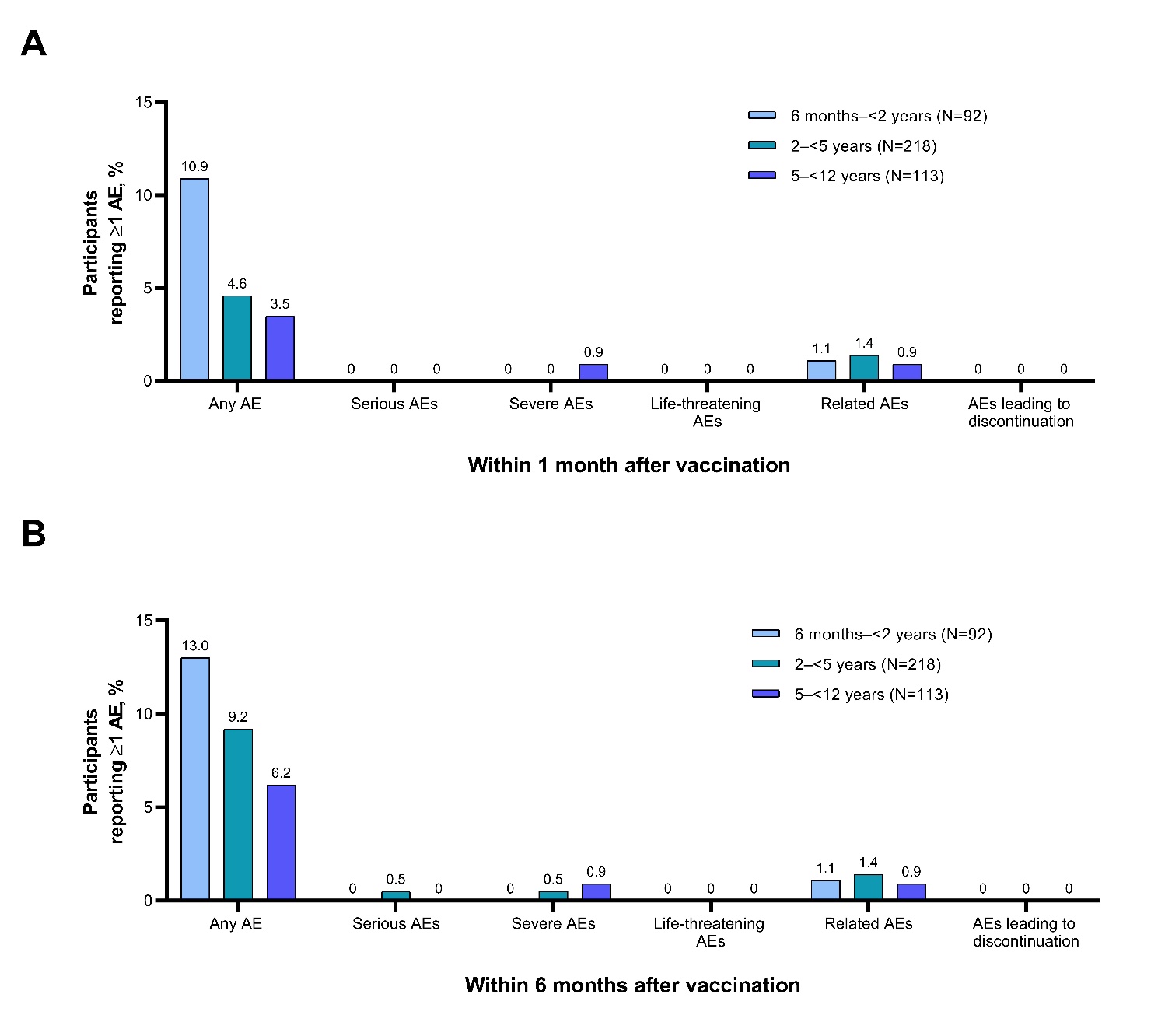

Supplement: piae062_suppl_Supplementary_Tables_1-9_Figures_1-3 [file piae062_suppl_supplementary_tables_1-9_figures_1-3.docx]
